# A Survey for Machine Learning-Based Control of Continuum Robots

**DOI:** 10.3389/frobt.2021.730330

**Published:** 2021-09-24

**Authors:** Xiaomei Wang, Yingqi Li, Ka-Wai Kwok

**Affiliations:** ^1^ Department of Mechanical Engineering, The University of Hong Kong, Pokfulam, Hong Kong, SAR China; ^2^ Multi-Scale Medical Robotics Center Limited, Hong Kong, Hong Kong, SAR China

**Keywords:** continuum robots, data-driven control, inverse kinematics (IK), kinematic/dynamic model-free control, learning-based control, machine learning, reinforcement learning, soft robots

## Abstract

Soft continuum robots have been accepted as a promising category of biomedical robots, accredited to the robots’ inherent compliance that makes them safely interact with their surroundings. In its application of minimally invasive surgery, such a continuum concept shares the same view of robotization for conventional endoscopy/laparoscopy. Different from rigid-link robots with accurate analytical kinematics/dynamics, soft robots encounter modeling uncertainties due to intrinsic and extrinsic factors, which would deteriorate the model-based control performances. However, the trade-off between flexibility and controllability of soft manipulators may not be readily optimized but would be demanded for specific kinds of modeling approaches. To this end, data-driven modeling strategies making use of machine learning algorithms would be an encouraging way out for the control of soft continuum robots. In this article, we attempt to overview the current state of kinematic/dynamic model-free control schemes for continuum manipulators, particularly by learning-based means, and discuss their similarities and differences. Perspectives and trends in the development of new control methods are also investigated through the review of existing limitations and challenges.

## 1 Introduction

Bioinspired by snakes, elephant trunks, and octopus tentacles, continuum robots are designed to structurally mimic their inherent dexterity and adaptability (Webster and Jones, 2010). In contrast to conventional rigid-link manipulators, “continuum” mechanisms leverage a series of continuous arcs without a skeletal structure to produce a bending motion (Robinson and Davies, 1999). Such design initially focuses on large-scale grasping, locomotion, and positioning in industrial applications (Robinson and Davies, 1999) or even urban search and rescue operations in confined environments (Jones and Walker, 2006a). The trade-off between high flexibility and low payload induces strict structural requirements. Gradually, with the reduced scale of continuum robots, the concerns are also diverted to the delicate steering of the slim robot body. The flexible characteristics of continuum robots are appropriate for surgical field applications. Enabling infinite degree-of-freedom (DoF) manipulations within small scales, continuum robots endow the target with flexible access and the patient with less invasion (Burgner-Kahrs et al., 2015). Moreover, pliable interventional devices with broad-range functions, such as catheters (Lee et al., 2018; Wang et al., 2018), afford much inspiration to the development of robotic continuum manipulators. Besides the mechanism of a robot, proper controllers and corresponding sensors are also necessary to guarantee accurate control performance.

**FIGURE 1:**
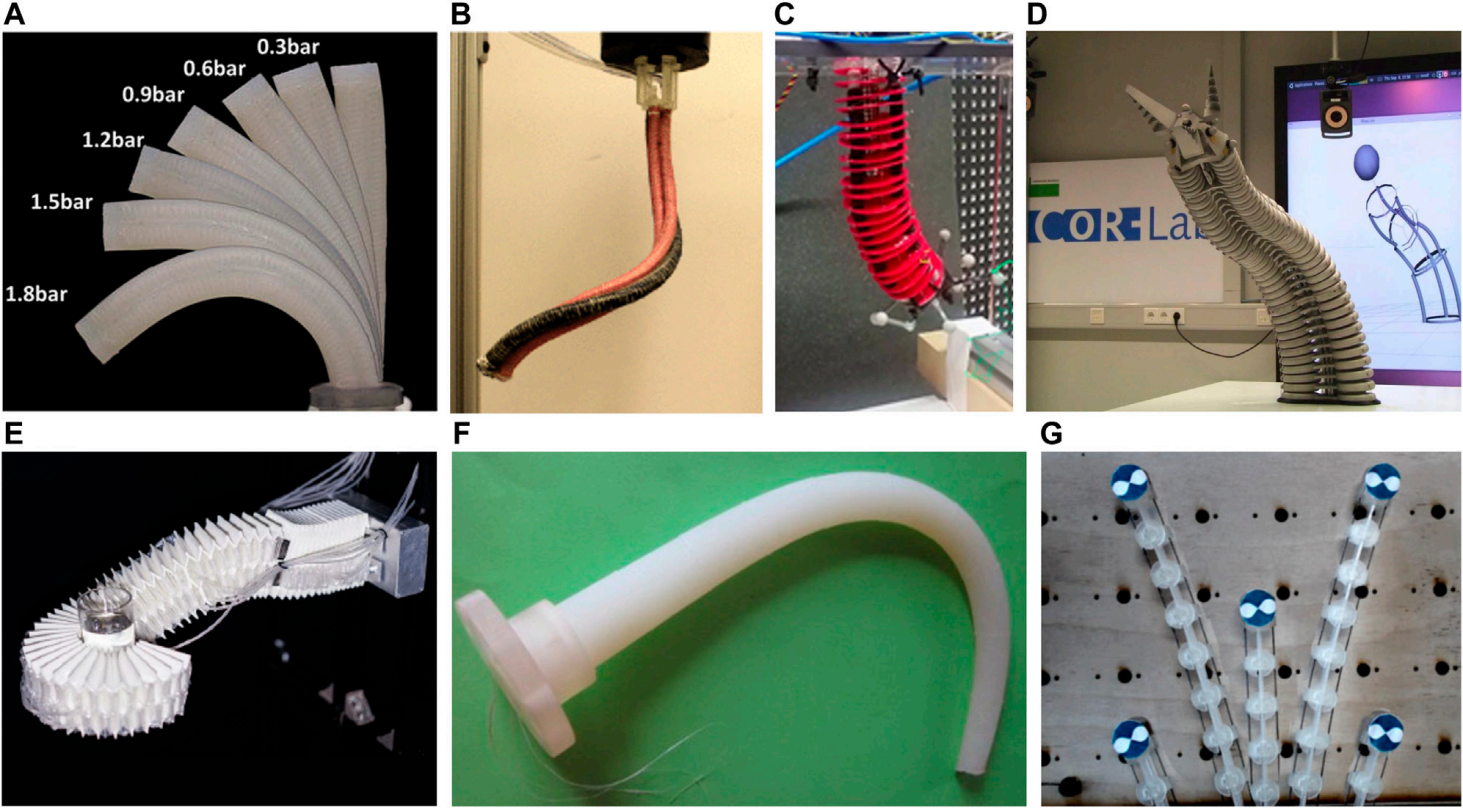
Continuum robots driven by **(A–E)** pneumatics or **(F-G)** tendons. Image sources for **(A)–(G)** in sequence: (Lee et al., 2017a; Satheeshbabu et al., 2019; Ansari et al., 2017a; Rolf and Steil, 2013; Jiang et al., 2016; Giorelli et al., 2015a; Yip and Camarillo, 2014).

Conventional rigid-link robots could be controlled directly by the commands of motors on each joint. Once the joint angles and the link lengths are available, the pose of all points, including the end-effector, can be fully determined. Different from rigid-link mechanisms which have definite kinematic mapping, soft robots meet great challenges in accurate analytical modeling, considering the nonlinear deformation induced by the actuation, material elasticity, and susceptibility to contacts with surroundings. Generally speaking, the actuation mechanism of continuum robots (Rolf and Steil, 2013; Yip and Camarillo, 2014; Giorelli et al., 2015a; Ansari et al., 2017a; Lee et al., 2017a; Satheeshbabu et al., 2019; Jiang et al., 2021) can be categorized as intrinsic and extrinsic (Robinson and Davies, 1999), based on the location of the actuator. The intrinsic mechanism means that the actuators are located inside and form as part of the mechanism (Fu et al., 2020). One example could be pneumatic-driven robots (Figures 1A–E), whose deformation is induced by the inflation of internal elastic chambers. Extrinsic mechanisms use external components to distort the robot body (Figures 1F,G), such as tendons/cables dragged by motors. Due to the high compliance of continuum manipulators, constraints imposed by obstacle interactions may deform the robot body into undesired shapes regardless of the actuation status. These effects are generally difficult to completely sense and feed back into the model, leading to unstable behaviors. In addition, the individual variation also leads to modeling uncertainties. For example, even with the same design prototype, different degrees of fabrication errors may require repetitive parameter tuning for all robots and their actuators (Nordmann et al., 2012). The characteristics of a specific robot may also change (e.g., wear-out effects) over the course of time.

Machine learning approaches provide a promising way out for the control of continuum robots. As the controller or inverse kinematic mapping is identified by experimental sensory data, people also title it as data-driven control. Sometimes, to distinguish it from the quantitative modeling which parameterizes the system using compact representations (e.g., differential equations) (Lunze, 1998), there were researchers using qualitative modeling to describe such circumstances (Melingui et al., 2014a; Melingui et al., 2014b). Apart from circumventing prior analytical modeling and computational complexity, another advantage of the learning-based control is the simplified requirement on sensors. Representative models include the (piecewise) constant-curvature (PCC)-based kinematic model (Jones and Walker, 2006b; Webster and Jones, 2010), Cosserat rod theory (Trivedi et al., 2008; Rucker et al., 2010; Giorelli et al., 2012), and spring-mass model (Kang et al., 2013) would require the measurements of actuator length/strain/curvature for more accurate use of the model, if not relying on the feedback. Their effectiveness in the feed-forward loop could not be guaranteed, since the analytic shape reconstructed from the set of possible length combinations or other configuration parameters is unknown and non-stationary (Nordmann et al., 2012). Fortunately, direct learning of the end-to-end mapping only needs the information of actuators (e.g., motor position/command) and a sensor that can capture the end-effector (or other control objectives like the shape) in the task space. Therefore, the burden for multiple types of sensors is alleviated and even eliminated.

Various machine learning techniques have the potential or have been validated for continuum robot control in existing works. In this article, we aim to summarize and discuss their representative implementations. Different from previous reviews such as the one by George Thuruthel et al. (2018) that provides the outlines of all possible control strategies, here machine learning–based approaches are intensively investigated with details and systematic analysis. Starting with the iterative machine learning methods, supervised learning methods follow as a main part. Since supervised learning techniques, which utilize known input and output datasets for mapping establishment, accord suitably with the thought of robot control, lots of research works have been conducted. Several examples showing the combination of learning-based and analytical models are also introduced. However, as semi-supervised learning and unsupervised learning are designed to learn classifications from small-portion tagged data and patterns from untagged data, respectively, few types have been applied in robot manipulation, which targets definite and accurate regression results. Although they are possible to implement in specific control units in the future, we will not discuss such categories in this article. Finally, the attempts at reinforcement learning on continuum robots are also elaborated upon for the first time. Reinforcement learning refers to a family of learning-based algorithms where the agent autonomously learns to deal with new tasks during the interaction with its environment. Compared to supervised learning where the model learns from the “answer key” in training data, reinforcement learning enables the model to discover the optimal behavior policy from experience. Nowadays, reinforcement learning applied in the control of soft robots has been attracting lots of interest and developing fast since it could avoid prior knowledge of robot configuration. Additionally, not limited to the modeling in a specific workspace, reinforcement learning is expected to extend the manipulation adaptivity to a complex and dynamic environment (Polydoros and Nalpantidis, 2017). At the end of this article, potential trends in the development of machine learning–based control methods are also discussed after a summary of existing works.

## 2 Iteration-Based Kinematic Model-Free Control

**FIGURE 2:**
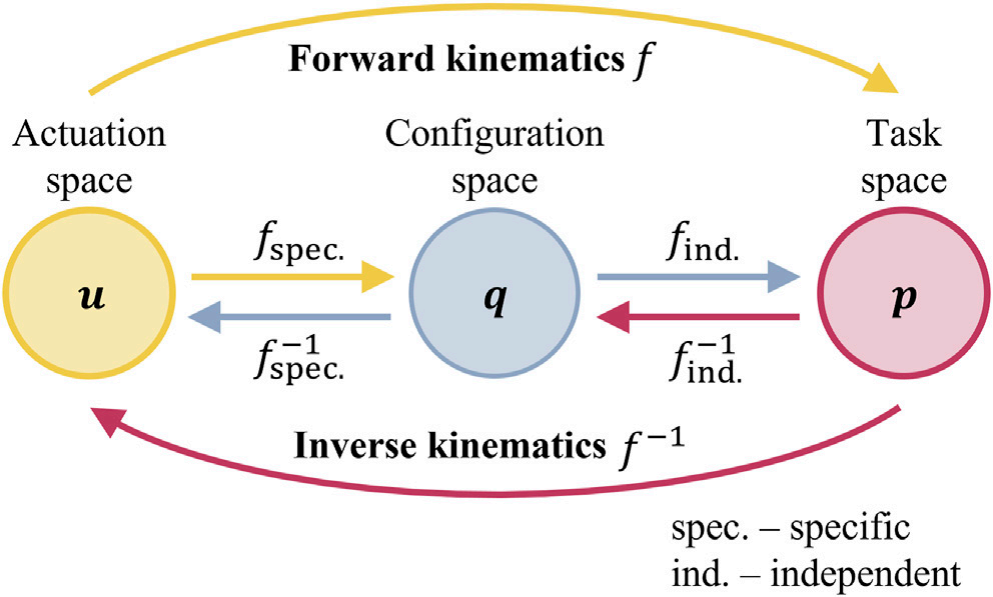
Mapping relationship between the three spaces (i.e., u : actuation space, q : configuration space, and p : task space) in soft robot control.

In model-based controllers, the kinematic model can be decomposed into two mappings (Jones and Walker, 2006b), namely, robot-independent mapping and robot-specific mapping. These mappings link three spaces separately. The robot-independent mapping depicts the relation between the configuration and task spaces, while the robot-specific mapping represents the relation between the actuation and configuration spaces (Figure 2). The task space means the feasible region of the control object (usually the workspace of the end-effector p ). The actuation space represents the command u on motors or other actuation types which could be instructed quantitatively. In rigid-link robots, the kinematic mapping could be definitely established between the actuation and task spaces, since the configuration space is mostly linearly related to the actuation space. However, in soft continuum robots, the configuration parameters characterizing the arc idealize the robot deformation by making several assumptions. The material elasticity or fluid dynamics would induce large nonlinearity between the actuation and configuration spaces, which is difficult to involve in the modeling. That is also why the actuation-related part is called robot-specific mapping. There are model-free approaches utilizing the idea of iterative optimization to refine the desired mapping relationship and conduct control. Some representative control theories also involve similar ideas, such as the model predictive control (Tang et al., 2019a). In the following sections, representative methods to iteratively learn the mapping or its inverse format will be introduced. The conventional control idea utilizes the inverse Jacobian matrix to build the mapping between the actuation and task spaces. Although the Jacobian matrix may vary nonlinearly during the robot motion, someone rationally hypothesized that such variations within a single control interval are minimal and linear, assuming the slow movement of continuum robots.

### 2.1 Optimization-Based Jacobian Matrix Estimation

Yip and Camarillo (2014), Yip and Camarillo (2016), and Yip et al. (2017) proposed a series of model-free closed-loop controllers based on the optimal control strategy. Different from deducing the Jacobian matrix regarding the analytical model (Siciliano and Khatib, 2016), one advantage of such methods is the quick initialization of the Jacobian matrix; during the execution of the robot, the Jacobian matrix can also be updated. Using the symbols in Figure 2, the actuator input (at equilibrium) is represented as $u\left(k\right)\in U^{m}$ at time step k , where $U^{m}$ denotes the m -dimensional actuation space, while the task space (e.g., the end-effector position in the Cartesian space $\in {\mathbb{R}}^{3}$ ) is denoted by p(k) . With the inverse Jacobian matrix, the discretized relationship from the task space to the actuation space can be as follows:

$$\text{Δ}u\left(k\right)=J^{-1}\text{Δ}p\left(k\right).$$ (1)

Assume that the robot actuation is designed as 3 DoFs which are independent from each other. The initialization of J can be finished by successively actuating the 3 DoFs in turn with an incremental amount $\text{Δ}u_{i}$ , i= 1, 2, 3 (i.e., the actuation commands are $\left[\begin{array}{ccc} \text{Δ}u_{1} & 0 & 0 \end{array}\right]$ , $\left[\begin{array}{ccc} 0 & \text{Δ}u_{2} & 0 \end{array}\right]$ , and $\left[\begin{array}{ccc} 0 & 0 & \text{Δ}u_{3} \end{array}\right]$ in turn), and measuring the corresponding displacements $\text{Δ}p_{i}$ , i= 1, 2, 3 . The initial Jacobian matrix could be constructed as follows:

$$J=\left[\begin{array}{ccc} J_{1} & J_{2} & J_{3} \end{array}\right],$$ (2)

where $J_{i}=\text{Δ}p_{i}/\text{Δ}u_{i}$ , i= 1, 2, 3 . The Jacobian matrix J is updated by quadratic programming as follows:

$$\begin{array}{l} \text{minimize}\;\;\;\;\;\;\;\;\;\;\;\;\text{||Δ}J\left(k\right)\text{||}\\ \text{subject to}\;\;\;\;\;\;\;\;\;\;\;\text{Δ}p\left(k\right)=J\left(k\right)\text{Δ}u\left(k\right)\\ J\left(k\right)=J\left(k-1\right)+\text{Δ}J\left(k\right) \end{array}$$ (3)

They also proposed to use force sensors to measure the tension of each tendon, therefore optimizing the actuation command with the minimal change. Besides the optimization-based construction of inverse kinematic mapping, machine learning–based methods have also been employed in various works, which are summarized in the following section.

### 2.2 Methods Utilizing Adaptive Kalman Filter

Li et al. (2017) considered the change of the Jacobian entries (forming system states $x\left(k\right)\in {\mathbb{R}}^{m\times n}$ of the robot) as the process noise of the stochastic system and used an adaptive Kalman filter to estimate the entries, avoiding the kinematic modeling procedure. The state and measurement (displacement of the robot end-effector $y\left(k\right)=\text{Δ}p\left(k\right)\in {\mathbb{R}}^{m}$ ) of the stochastic system are constructed as follows:

$$\begin{array}{c} x\left(k+1\right)=x\left(k\right)+\eta \left(k\right)\\ y\left(k\right)=H\left(k\right)x\left(k\right)+\delta \left(k\right), \end{array}$$ (4)

where the block-diagonal measurement matrix $H\left(k\right)=\mathrm{diag}\left(\text{Δ}u{\left(k\right)}^{T},m\right)\in {\mathbb{R}}^{m\times nm}$ is composed by the change of actuation pressure/voltage $\text{Δ}u\left(k\right)=u\left(k\right)-\text{Δ}u\left(k-1\right)\in {\mathbb{R}}^{n}$ ; The process and measurement noises are represented by η(k) and δ(k) , respectively, which are assumed to be Gaussian noises with covariance matrices of Q and R . The two-step recursive formulas contribute to the calculation of the Jacobian matrix by the following:

$$\begin{array}{c} {\hat{x}}_{p}\left(k+1\right)=\hat{x}\left(k\right)+\eta \left(k\right)\\ P_{p}\left(k+1\right)=P\left(k\right)+Q\left(k\right), \end{array}$$ (5)

and

$$\begin{array}{c} K\left(k\right)=P_{p}\left(k\right)H^{\text{T}}\left(k\right){\left[H\left(k\right)P_{p}\left(k\right)H^{\text{T}}\left(k\right)+R\right]}^{-1}\\ P\left(k\right)=\left[I-K\left(k\right)H\left(k\right)\right]P_{p}\left(k\right)\\ \hat{x}\left(k\right)={\hat{x}}_{p}\left(k\right)+K\left(k\right)\left[y\left(k\right)-H\left(k\right){\hat{x}}_{p}\left(k\right)\right] \end{array}$$ (6)

where ${\hat{x}}_{p}\left(k\right)$ and $P_{p}\left(k\right)$ are separately the predicted state estimation and error covariance, their corresponding updates are $\hat{x}\left(k\right)$ and P(k) . The optimal Kalman gain is K(k) . The detailed calculation approach can be found in the study by Li et al. (2017), where the use of an adaptive covariance matrix for the process noise η(k) improves the filter’s convergence and tracking performance against system uncertainties. Based on the updated Jacobian matrix, an optimal vector is further implemented into the controller to determine the appropriate configuration allowing for the lowest deformation of the robot (i.e., minimal actuation variation).

## 3 Supervised Learning of Inverse Statics/Kinematics

This section will summarize the methods to learn the desired mapping in control offline, that is, using learning-based algorithms to approximate the statics/kinematics of the entire mapping from the actuation space to the task space. Here, the difference between statics models and kinematics models is briefly explained. A statics model depicts the robot configuration by assuming all forces on the manipulator at rest under equilibrium conditions. A kinematics model describes the robot motion only based on the geometric relationship, without considering the applied forces. For example, in the case of soft cable-driven manipulators, the direct forward statics model maps the cable tensions onto the tip position, while the inverse statics model calculates the cable tensions in order to make the tip on the desired position (Giorelli et al., 2013a; Thuruthel et al., 2016b). It is an absolute mapping scheme. The kinematics can be regarded as an approach to represent relative/incremental movements, where the change from the current robot status to the desired one is focused, or it can also be described as a mapping between the actuator velocity and the end-effector velocity. Accurate inverse statics can be implemented in open-loop control, and the inverse kinematics can be coordinated with the feedback in closed-loop control. As the most straightforward applications of learning-based algorithms, there are lots of articles from 2013 to 2020 (Giorelli et al., 2013a; Giorelli et al., 2013b; Melingui et al., 2014a; Giorelli et al., 2015a; Giorelli et al., 2015b; Chen and Lau, 2016; Thuruthel et al., 2016b; Thuruthel et al., 2016a; Lee et al., 2017a; Xu et al., 2017; Ho et al., 2018; Fang et al., 2019; Bern et al., 2020) utilizing this kind of thought to handle the continuum robot control. Details of these works are summarized in Table 1. For the convenience of showing their similarities and conducting systematic comparisons and discussions, we organized the following sections: Section 3.1 and Section 3.2.

**TABLE 1:** Sampling of inverse statics/kinematics learning-based control for continuum robots. Intended to be exemplary, not comprehensive.

Literature	Classification criteria							
	Model	Robot structure	Actuation (length)	Act. & TaskDoFs	Mapping to be learned	Samples	Task	Accuracy/mm (mean/STD/max)
Giorelli et al.	1-hidden-layer FNN							
a. Giorelli et al. (2013a)	a. 21 neurons	Silicone conical robot	Tendon (310 mm/280 mm)	a. 2 & 2	$u\left(k\right)=f_{s}^{-1}\left(p*\left(k\right)\right)$	500 (8:2)	Simulation: path generation. Discrete points	2.27/1.70/9.30
b. Giorelli et al. (2013b)	b. 34 neurons			b. 3 & 3		500 (8:2)		4.2/2.8/12.3
c. Giorelli et al. (2015a)	c. 6 neurons			c. 2 & 2		405 (8:2)		22.88/11.80/59.79
d. Giorelli et al. (2015b)	d. 28 neurons			d. 3 & 3		395 (8:2)		7.35/--/22.22
Melingui et al. (2014a)	Forward: FNN. Inverse: 1-hidden-layer NN in DSL	CBHA	Pneumatic (NA)	^a^3 × 2 & 3	Forward: $p\left(k\right)=f_{s}\left(u\left(k\right)\right)$	4,096 (7:1.5:1.5)	Comparison: robot and model postures (under the same actuation)	Inverse: 1.1 e^−4^ (MLP) ∼ 4.1 e^−4^ (RBF)/--/--
					Inverse: $u\left(k\right)=f_{s}^{-1}\left(p*\left(k\right)\right)$			
Thuruthel et al.	1-hidden-layer NN.							
a. Thuruthel et al. (2016a)	a. 20 neurons	a. BHA	a. Pneumatic (0.9 m)	a. 3 × 3 & 3	$q\left(k\right)={\hat{f}}_{s}^{-1}\left(q\left(k-1\right),p*\left(k\right)\right)$	a. 10,000 (7:3)	Simulation: continuous path following in terms of position (P)/orientation (O)	*p*: <1/1.504/--
b. Thuruthel et al. (2016b)	b. 40 neurons	b. Silicone conical	b. Tendon (31 cm)	b. 12 & 6	$\text{Δ}q\left(k\right)={\hat{f}}_{k}^{-1}\left(q\left(k-1\right),\text{Δ}\theta *\left(k\right)\right)$	b. 14,000 (8:2)		*p*: 8.5/2.8/-- O/°: 3.21/1.71/--
Chen and Lau (2016), Xu et al. (2017)	ELM, GMR, or KNNR	Silicone serpentine	Tendon (NA)	^a^2 & 2	$u\left(k\right)=f_{s}^{-1}\left(p*\left(k\right)\right)$	20,000	Simulation & real robot: path following	2.1275∼ 2.5556/−/−
Lee et al. (2017a)	LWPR *online update*	Silicone cylindrical	Pneumatic (93 mm)	3 & 2	$\text{Δ}u\left(k\right)=f_{k}^{-1}\left(n\left(k-1\right),u\left(k-1\right),\text{Δ}n*\left(k\right)\right)$	>1,000 for initialization [FEA Lee et al. (2017b)]	Angular path following (2D) + external forces	Free space/°: 0.90/0.65/2.80
								Disturbed/°: 2.49/1.74/11.03
Ho et al. (2018)	LWPR *online update*	Silicone cylindrical	Pneumatic (155 mm)	3 × 2 & 3	$\text{Δ}u\left(k\right)=f_{k}^{-1}\left(u\left(k-1\right),\text{Δ}p*\left(k\right)\right)$	—	Path following (3D) + tip load (72% robot mass)	With load: 0.98/0.26/--
Fang et al. (2019)	LGPR *online update*	Silicone cylindrical	Pneumatic (67 mm)	3 & 2	$\text{Δ}u\left(k\right)=f_{k}^{-1}\left(u\left(k-1\right),\text{Δ}z*\left(k\right)\right)$	300	Path following (2D visual servo) + tip load	Free space/pixel: 5.4/--/11.5
Bern et al. (2020)	a. 2-hidden-layer FNN (20 × 2)	Silicone cylindrical	Tendon (20 cm)	a. 3 & 2	$u\left(k\right)=f_{s}^{-1}\left(p*\left(k\right)\right)$	a. 308 (9:1)	a. Real robot	Real robot: 6.2∼9.2/−/−
	b: 3-hidden-layer FNN (25 × 3)			b. 3 × 2 & 2		b. 15414 (8:1:1)	b. Simulation 2D path following	—

a Only the actuation dimensions that are related to the end-effector control are considered; “×2” or “×3” means the number of segments in the manipulator.

(F)NN, (feed-forward) neural network; GMR, Gaussian mixture regression; FEA, finite element analysis; DSL, distal supervised learning; KNNR, *K*-nearest neighbors regression; STD, standard deviation; (C)BHA, (compact) bionic handling assistant; LWPR, locally weighted projection regression; MLP, multilayer perceptron; ELM, extreme learning machine; LGPR, locally Gaussian process regression; RBF, radial basis function.

### 3.1 Mapping to Be Learned

As in Figure 2, successive mappings between the actuation and configuration spaces, the configuration and task spaces, or directly from the actuation space to the task space have to be defined as the forward kinematics. The actuator input (at equilibrium) is represented as $u\left(k\right)\in U^{m}$ at time step k , where $U^{m}$ denotes the m -dimensional actuation space. Let q(k) be the manipulator configuration parameters under input u(k) , which corresponds to a specific task space status such as the end-effector position $p\left(k\right)\in {\mathbb{R}}^{3}$ and the orientation normal $n\left(k\right)\in {\mathbb{R}}^{3}$ in the Cartesian space. The collective pose variable $\theta \left(k\right)=[p\left(k\right),n\left(k\right)]\in {\mathbb{R}}^{6}$ can be thus denoted. It should be noticed that for continuum robots, not only can the pose of an appointed point be defined as the control objective but also the entire manipulator shape can be represented by more DoFs. Here, we take the three-dimensional (3D) position p(k) as an example to show the representation of the absolute forward robot-independent mapping as follows:

$$p\left(k\right)=f_{ind.}\left(q\left(k\right)\right),$$ (7)

and that of absolute forward statics as follows:

$$p\left(k\right)=f_{s}\left(u\left(k\right)\right).$$ (8)

With quasi-static movements, the forward transition model can be expressed in the incremental format as follows:

$$\text{Δ}p\left(k\right)={\hat{f}}_{k}\left(q\left(k-1\right),\text{Δ}u\left(k\right)\right),$$ (9)

where Δu(k)=u(k)−u(k−1) is the difference of inputs between time step k and (k+1) , and Δp(k)=p(k)−p(k−1) denotes the incremental displacement. The control objective of inverse statics is to generate an actuation command u(k) , thus steering the manipulator to the desired p*(k) in the task space as follows:

$$u\left(k\right)=f_{s}^{-1}\left(p*\left(k\right)\right).$$ (10)

In inverse kinematics for closed-loop control, the change of actuation command Δu(k) , thus achieving the desired movement Δp*(k)=p*(k)−p(k−1) in the task space, is calculated. Therefore, mapping Eq. 11 is deduced to approximate the inverse kinematics of Eq. 9, as follows:

$$\text{Δ}u\left(k\right)={\hat{f}}_{k}^{-1}\left(q\left(k-1\right),(p*\left(k\right)\right).$$ (11)

The inverse transition ${\hat{f}}_{s}^{-1}$ heavily depends on the last robot configuration q(k−1) that is supposed to be unknown during the training of an operation. However, since during quasi-static movements, the robot configuration q(k−1) can be represented/defined by the corresponding actuation u(k−1) , the inverse kinematics function Eq. 11 can be approximated as follows:

$$\text{Δ}u\left(k\right)=f_{k}^{-1}\left(u\left(k-1\right),\text{Δ}p*\left(k\right)\right).$$ (12)

here, as long as the sensory information of the task space variable p and the encoded actuation command u are available, the inverse kinematics can be learned to accomplish various control tasks, without the need of analytical/quantitative modeling. According to the control object, the task space can be specified as the 2D/3D position p(k) , the 2D/3D orientation n(k) , or be defined in other coordinate frames. For example, in the visual-servoing tasks (Fang et al., 2019), the position of the end-effector z(k) in the 2D camera frame/view is desired to stay focused or follow paths. For some tasks like whole-arm grasping, the entire robot body should be considered and commanded.

### 3.2 Learning Approaches

As can be seen from Table 1, neural networks (NNs) are the most commonly used regression model to approximate the mapping. The feed-forward NN (FNN) is the basic type, where the information always flows from the input side to the output side with the weighted calculation of hidden layers (Svozil et al., 1997). If not specified, an NN usually indicates an FNN. Specific types of FNNs like the extreme learning machine (ELM) were also applied. Besides, some regression methods perform satisfying results in continuum robot control, and representative ones can be the locally weighted projection regression (LWPR) (Lee et al., 2017a; Ho et al., 2018) and (locally) Gaussian process regression (GPR) (Fang et al., 2019). It can be found that FNNs are adaptive to find the absolute relationships (Giorelli et al., 2013a; Giorelli et al., 2013b; Melingui et al., 2014a; Giorelli et al., 2015a; Giorelli et al., 2015b; Thuruthel et al., 2016a; Chen and Lau, 2016; Xu et al., 2017; Bern et al., 2020), for example, forward and inverse statics relations as in Eq. 8 and Eq. 10. Such cases correspond to open-loop controls, where online sensory devices are not available. For the learning of relative mappings (Thuruthel et al., 2016b; Lee et al., 2017a; Ho et al., 2018; Fang et al., 2019) such as Eq. 9 and Eq. 11, regression-based methods usually guarantee more stable convergence. Combined with corresponding sensing feedback, these mappings enable higher accuracy since the control loop can be closed. Most of the models were confirmed in advance using selected training samples. Related discussions of the data exploration can be found in Section 3.3.1. Regression models also support the online update using newly collected sensory data, but the trade-off will be the calculation load. For example, GPR requires the matrix inversion calculation (the matrix dimension is related to the sample amount) for each time of model refinement; therefore, the sampling window could not be too large. Locally, GPR models were therefore implemented in the study by Fang et al. (2019), where the whole workspace can be divided into several sections (≤300 samples for each) for independent model training and updating. Such *k*-means–guided localization could guarantee high computation speed (>20 Hz).

### 3.3 Problems to Be Considered

#### 3.3.1 Data Exploration

For the offline trained models, collection and selection of the training data are crucial for the accuracy of the model. Requirements of the samples are as follows: 1) covering the whole workspace of the robot end-effector and 2) evenly distributed in the task space to ensure consistent estimation performance in all areas. Most existing works used a kind of motor babbling approach (Thuruthel et al., 2016b), applying the interpolation in the actuation or configuration space. The number of optimal samples will be dependent on the workspace range and motion step size. Sample (input–output) pairs were collected by incrementally actuating the motors (or other specific mechanisms) with a fixed increment and saving the corresponding end-effector status. With the actuation command in the safe range, this procedure will result in an ergodic dataset. De-noising and filtering were usually needed to abstract high-quality and nonrepetitive samples. To fully exploit the advantage of learning-based approaches, normalizations of the input and output data (into [−1,1] or [0,1]) were conducted before training. For the training of relative mappings, an additional process will be finding out all possible displacements between two random points and filtering out motions in a fixed range of step size (Fang et al., 2019). Samples were usually divided into training, testing and validation sets, where the training dataset usually occupies more than 70% of them. The concept of goal babbling (Rolf et al., 2010) was also proposed but more like a standalone control approach rather than a means of data exploration. It depended on an iterative bootstrapping and refinement procedure of the inverse kinematics estimates, by a path-based sampling approach. The training data were generated along paths (a set of linearly interpolated target points) *via* repeating the “trying to reach” process. This thought has been validated on the simulation of a bionic handling assistant (BHA) robot (Rolf and Steil, 2013).

#### 3.3.2 Structural Optimization of Neural Networks

Although no prior knowledge of the robot modeling is required in data-driven approaches, there are hyper-parameters to be tuned, especially in the NNs, since LWPR and LGPR would not require the manual tuning of any hyper-parameters. The structural optimization of NNs focused on the number of hidden layers and neurons once a specific model was selected. Previous works of Giorelli et al. (2013a), Giorelli et al. (2013b), Giorelli et al. (2015a) and Bern et al. (2020) discussed the heuristic procedure. They observed that small-size networks usually had poor performances, which may not be expressive enough to capture the robot’s physical characteristics. Increasing the size of the network yields greater accuracy; however, it may encounter a bottleneck or even induce a new trend of accuracy decrease once past a certain size. A hypothesis is that the gathered dataset was insufficiently large to properly train such a large NN. Overfitting is also a factor to be avoided. Considering the actuation DoFs, one hidden layer would be enough for single-segment robots, while the multi-segment redundant manipulators may require a similar increasing number of hidden layers.

#### 3.3.3 Actuation/Configuration Redundancy

In the learning-based kinematic control, redundancy is the feature which is possible to generate inconsistent samples with the same effector pose but different joint angles or actuation commands. Learning from such examples will lead to invalid solutions (Lunze, 1998). The redundancy may result from the configuration space, that is, the robot-independent mapping (the configuration space to the task space) is redundant. This case exists in the multi-segment continuum robots, where the actuation space is 6D or 9D, but the task space only involves 3D positional information. Another cause of redundancy may derive from the actuation space, and this is a specific circumstance in tendon-driven or pneumatic-driven mechanisms. As sometimes the overall elongation change can be ignored, the relationship from the actuation space to the configuration space (robot-specific mapping) is also a multiple-to-single mapping. Both kinds of problems had been discussed and considered in several literatures. One way to resolve the redundancy in the actuation/configuration space is manually biasing the original training data to only allow a single inverse solution (Fang et al., 2019). This method is an optimal way out in single-segment manipulator control, since it can reduce the unnecessary complexity of training data without reducing the robot flexibility. However, for kinematically redundant (configuration-redundant) manipulators, the benefits of using multi-segment/DoFs are mostly lost and will result in improperly accomplished tasks (Peters and Schaal, 2008). For such manipulators, the alternative method is to introduce a reward/cost function to draw the system to a desired solution (Mahler et al., 2014). This is the most widely accepted approach, and an example can be found in the study by Ho et al. (2018), where the task was formulated as a constrained optimization problem. However, drawbacks of the cost function using the sum of the squared error Giorelli et al. (2013b) were raised in the study by Melingui et al. (2014b), pointing out that it is possible to yield nonconvex inverse mappings. They solved it by implementing a squared penalty term in the objective function of the inverse NN, thus selecting one particular inverse model from the redundancy manifold. In addition, the weighting scheme (Nordmann et al., 2012) and method taking both/all-segment trajectories into account (Melingui et al., 2015) had also been tested.

### 3.4 Combination of Analytical Model and Learning-Based Component

Either in general or specific tasks, there are hybrid controllers proposed, combining the analytical dynamics/kinematics model and learning-based approaches (e.g., NN) to accomplish robust control performance. Methods combining the learning-based and conventional components also appeared in several works. For example, Braganza et al. (2007) proposed a framework comprising a neural network feed-forward component and a nonlinear feedback component, where the NN based on augmented back propagation was utilized as a feed-forward compensator for the nonlinear uncertain dynamics. The proposed scheme enables continuous and asymptotic tracking without any prior knowledge of the robot dynamic model, and the back propagation technique enables fast online training of the NN weight matrices referring to the tracking error signal. Similarly, Queißer et al. (2014) presented the idea to superimpose a learning-based inverse equilibrium dynamics model for the feed-forward control and then combined it with a feedback controller. Subudhi and Morris (2009) implemented a hybrid fuzzy neural control (HFNC) scheme for a multi-link flexible manipulator. The control actions were determined by both a fuzzy controller (the primary loop) and an NN controller (the secondary loop) to compensate for the coupling effects. Tang et al. (2019a) and Tang et al. (2019b) proposed a control framework combining the model-free iterative learning (Rong-Hu and Zhong-Sheng, 2007) and model predictive control for trajectory-tracking control of a wearable soft robotic glove. The integration of the kinematic model and the machine learning trained model was also validated, and most of the learning-based parts acted as an error compensator of the analytical model, such as in the works of Reinhart and Steil (2016) and Reinhart et al. (2017).

## 4 Reinforcement Learning Strategies

With the development of artificial intelligence, reinforcement learning is emerging in the robotics community, which is a natural application for learning-based control since the interaction between robots and the environment is necessary. Reinforcement learning offers appealing tools enabling to complete sophisticated tasks and accommodate complex environments, which may be limited in conventional control strategies.

Reinforcement learning is regarded as a Markov decision process (MDP), represented using a tuple (S,A,p(s’|s,a),R,γ) *.* In the agent’s interaction with the environment, S is the set of the agent’s possible states, where s is the current state and s’ is the next state after the agent transition. A presents the set of the agent’s actions, where a is the action. p(s’|s,a) is the state-transition probability of the agent transiting from the current state s to the future state s’ after the implementation of action a . The states and actions constitute the trajectory $\tau =\left(s_{0},a_{0},s_{1},a_{1},\ldots \right)$ . R(s, a) defines the reward function after the agent executes action a at state  s , and for convenience, r(s,a) represents the immediate reward of one transition and R is the accumulated reward or expected return of the whole trajectory, as written in Eq. 13.

$$R\left(\tau \right)=\sum_{t=0}^{T}r\left(s_{t},a_{t}\right),$$ (13)

where T is the number of time steps in the trajectory τ . For the infinite-horizon reinforcement learning problem, the effect of a future reward on the present decision could be considered with the reward discount factor γ ranging from 0 to 1, which is common for classical reinforcement learning (Kober et al., 2013). The accumulated reward is written as follows:

$$R\left(\tau \right)=\sum_{t=0}^{\infty }\gamma^{t}r\left(s_{t},a_{t}\right).$$ (14)

For the finite-horizon reinforcement learning problem, the average reward function is considered as shown in Eq. 15.

$$R\left(\tau \right)=\frac{1}{T}\sum_{t=0}^{T-1}r\left(s_{t},a_{t}\right).$$ (15)

Policy π(a|s) is the mapping from the state s to the action a , namely, given the current state, it could suggest the next step to obtain an optimal reward. The value function could evaluate the quality of the policy, offering the quantitative metric for the behavior decision maker. One of the value functions is called the state–value function $V^{\pi }\left(s\right)$ , which defines the value of a state s  under the policy π .

$$V^{\pi }\left(s\right)=E_{a∼\pi }\left[R\left(\tau \right)|s_{t}=s\right].$$ (16)

Another one is the action–value function $Q^{\pi }\left(s,a\right)$ , which could assess the action a at state s under the policy π .

$$Q^{\pi }\left(s,a\right)=E_{a∼\pi }\left[R\left(\tau \right)|s_{t}=s,a_{t}=a\right].$$ (17)

Using Eq. 13 and Eq. 14 and the Bellman equation (Sutton and Barto, 2018), value functions could be written as follows:

$$V^{\pi }\left(s\right)=\sum_{a}\pi \left(a|s\right)\left[r\left(s,a\right)+\gamma \sum_{s^{t}}P\left(s^{\prime }|s,a\right)V^{\pi }\left(s\prime \right)\right],$$ (18)

$$Q^{\pi }\left(s,a\right)=r\left(s,a\right)+\gamma \sum_{s^{\prime }}P\left(s^{\prime }|s,a\right)V^{\pi }\left(s\prime \right).$$ (19)

### 4.1 The Goal of Reinforcement Learning

In the context of mathematics, the goal of reinforcement learning is to explore an optimal policy that could instruct actions based on the present observation. The objective is to maximize the accumulated reward (Eq. 13), which determines the learning task. When considered in robot control, the goal of reinforcement learning is to figure out a control strategy that could generate optimal instruction for robot action in order to accomplish the specified task effectively. The reward function is designed manually to train the robot with certain characteristics, for example, penalizing the times of transition to enable the robot to reach the target in as few movements as possible.

**FIGURE 3:**
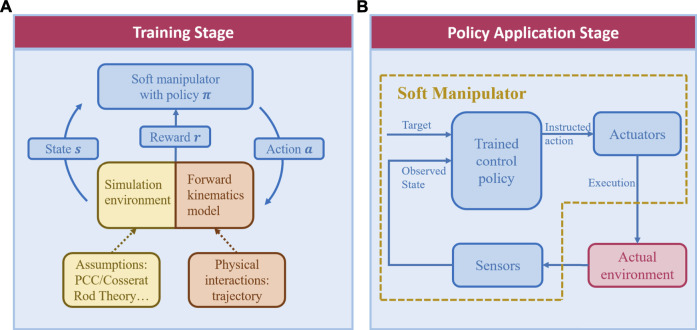
Schematics of reinforcement learning in soft robot manipulation, with **(A)** the policy training stage and **(B)** the application stage separately shown.

For instance, there is a soft planar robot planned to touch a designated point in 2D space, where reinforcement learning can be explained as below. Sensors on the robot provide the observation about its relative position to the target, as well as moving velocity and direction, which describe the current state s . The soft robot is actuated using several inflating air chambers so that it could elongate or contract, thus steering the robot to the left and the right, indicating the action set A . Reward function R(s, a) is designed manually, for example, the reward on short relative distance and the penalty on transition times could accelerate the learning convergence process. Policy π(a|s) gives the action suggestion based on the observation of the current state to maximize the cumulative reward. Figure 3 describes the pipeline of reinforcement learning algorithms in soft manipulator control. In the training stage (Figure 3A), trajectories calculated from the forward kinematic model or the simulation environment contribute to training the control policy. In the application stage (Figure 3B), every time upon receiving the sensor-observed state and target information, the learned control policy would give instructed actions, which would be executed by the actuator. Specificities of the application in continuum robots will be introduced in the following section.

### 4.2 Reinforcement Learning in Soft Robot Manipulation

Compared with the conventional joint-linked robots, the application of reinforcement learning in soft robots may face a number of challenges requiring specific attention. Reinforcement learning enables robots to learn from experience, which demands thousands of interactions with the environment. In addition to the tedious data collection, the noisy data, incomplete observation in practice, and the highly frequent movement could even damage the soft robot since it is mainly actuated hydraulically or pneumatically. To improve the effectiveness of training data collection, the model-free reinforcement learning approach has arisen, which obtains the learning experience from simulations. However, when transferred from the simulation to the prototype, the model-free algorithm might perform with large deviations since the modeling without real data could be inaccurate. Besides, the compliance and flexibility of soft robots cause high dimensional and continuous space, as to which the appropriate state and space discretization methods are expected in reinforcement learning. Furthermore, it is noticed that the soft manipulator would experience attenuation when exerted by external loads or disturbances. Not only could it impede the modeling of robots but it also makes the loading robustness experiment a necessity for the validation of reinforcement learning.

Referring to various criteria, reinforcement learning could be classified into different categories, such as model-based/model-free, policy-based/value-based, and averaged/discounted return function algorithms. The subsequent sections will introduce the first two kinds of categories in detail.

#### 4.2.1 Model-Based Vs. Model-Free Reinforcement Learning

Upon the employment of explicit transition functions, reinforcement learning could be classified into two categories: model-based and model-free algorithms. In robotic applications, model-based methods mostly concentrate on forward kinematics/dynamics models which require prior knowledge on robots or environments (Moerland et al., 2020). Unlike the joint-linked robots soft robot modeling relies on data-driven methods or geometric assumptions to establish the forward model. Subsequently, the forward kinematics/dynamics model could generate robot trajectory data for policy optimization.

Model-free reinforcement learning would exploit the virtual training data for policy learning. That is, the data are obtained *via* robot simulations. It is worth noting that the modeling of the robot and the environment in the simulation is based on simplified assumptions such as PCC (Webster and Jones, 2010; Lee et al., 2017a) and Cosserat rod theory (Antman, 2005) rather than the real interaction data, and such modeling would not be counted as a model-based approach. In the following sections, for the sake of clear expression, methods with kinematics/dynamics model denote model-based reinforcement learning; while those without such model represent model-free approaches.

##### 4.2.1.1 Reinforcement Learning With Kinematics/Dynamics Model

The policy trained on kinematics/dynamics model-based methods can perform stably, while in model-free methods, the derivation may be large when the algorithm is implemented from the simulation into the real robot. This is reasonable as the data from physical interaction are more realistic and persuasive than the one from simulation, where the virtual model is established using many simplifications and assumptions. Moreover, when the robot interacts with the environment in a circumstance that never appeared before, the policy might be invalid.

Thuruthel et al. (2018) leveraged the model-based policy learning algorithm on a simulated tendon-driven soft manipulator capable of touching dynamic targets. A nonlinear autoregressive network with exogenous inputs (NARX) was employed to establish the forward dynamic models using the observed data. Policy iteration from the sampled trajectories was used to give the optimal action directly. Wu et al. (2020) accomplished the position control of a cable-driven soft arm employing Deep Q-learning. Similar to the procedure in the study by Thuruthel et al. (2018), experiment data was collected to model the manipulator in simulation.

However, the shortcoming of kinematics/dynamics model-based reinforcement learning is that the physical interactions would be time-consuming and the data may be noisy; they might even bring more mechanical wear on the robot prototype, particularly the vulnerable soft robot (Polydoros and Nalpantidis, 2017). As a result, the approach not requiring physical data has drawn lots of attention from soft robot researchers.

##### 4.2.1.2 Reinforcement Learning Without Kinematics/Dynamics Model

In recent years, kinematics/dynamics model-free reinforcement learning implemented on the physical robot has attracted interest in the soft robot community, since it could circumvent the accuracy requirement of analytical modeling, which is hampered by the intrinsic nonlinearity and uncertain external disturbances. In general, the trajectory data are generated in the simulation environment for model-free reinforcement learning, and subsequently, the trained model would be transferred to the physical robot.

You et al. (2017) investigated a model-free reinforcement learning control strategy for a multi-segment soft manipulator, called honeycomb pneumatic networks (HPNs), capable of physically reaching the target in 2D space. The control policy was learned using Q-learning with the simulation data, demonstrating its effectiveness and robustness in simulation and practice. Jiang et al. (2021) adopted the same soft arm and developed a hierarchical control algorithm for complex tasks such as opening a drawer and rotating a handwheel. The control architecture was inspired by human decision-making process. The system consisted of three levels, sequentially low-level motion controller, high-level behavior controller and behavior planner. Q-learning was implemented in the low-level motion controller. The research demonstrated the feasibility of a relatively simple control algorithm on interaction tasks in unstructured environments. Considering that simple Q-learning cannot handle the high-dimension workspace and action of soft robots, Deep Q-learning which leverages the advantage of deep learning to learn policy is prevailing in continuous control. Satheeshbabu et al. (2019) proposed an open-loop position control strategy for a spatial soft arm named BR^2^ based on Deep-Q Network (DQN). Cosserat rod formulation–based simulation is used for training data generation. The positioning accuracy (>94%) in simulation and the real environment, even with the external load, indicated that the control approach was effective and robust. You et al. (2019) introduced a control strategy enabling the soft catheter to move in a heart model using Dueling DQN (DDQN). This algorithm defines the Q-value as the summation of the state value and the advantage function. Isolating the value enables more precise state approximation and higher learning efficiency (Wang et al., 2016).

Ansari et al. (2017b) exploited model-free multi-agent reinforcement learning (MARL) on a soft arm to complete an assistive bathing task, where each actuator of the manipulator is regarded as an agent, sharing the common environment. The actor-critic algorithm consists of two parts. The actor performs as policy π, accepting the current state, thus generating the next action. The critic assesses the state-action tuple through the state-value function. In the study by Ansari et al. (2017b), the state-action-reward-state-action (SARSA) algorithm was adopted as the critic to evaluate the policy. In contrast to Q-learning, SARSA employs the same policy to sample and optimize, which is an on-policy learning algorithm. In the study by Satheeshbabu et al. (2020), the BR^2^ soft continuum arm was improved with vision feedback and deep deterministic policy gradient (DDPG), which is a family of actor–critic algorithms. Compared to the previous open-loop control scheme, the closed-loop control method could not only decrease the error obviously but also enable the soft manipulator to track the relatively complex curve path. This research indicates that state feedback and closed-loop control could offset the real-world derivation in model-free reinforcement learning. Liu et al. (2020) presented a control strategy incorporating proximal policy optimization (PPO) and a central pattern generator (CPG) for soft robot snakes, which could track the planar changing goals. Comparison of training steps/time among these different tasks can be found in Table 2.

**TABLE 2:** Sampling of reinforcement learning control for continuum robots, including the algorithm, task, and training duration. Intended to be exemplary, not comprehensive.

Literature	Reinforcement learning algorithm	Task	Training steps/time	Distance error/success rate
Thuruthel et al. (2018)	Policy search	3D position reaching	8,000 s	Without load: 0.009∼0.017 m
				With load: 0.022 m
Wu et al. (2020)	Q-learning	2D position reaching	1,000 iterations	Without load: <0.5 cm
				With load: <1 cm
You et al. (2017)	Q-learning	2D position reaching	1,000 iterations	<10 mm
Jiang et al. (2021)	Q-learning	Interaction tasks including drawer opening and handwheel rotating	120 iterations (about 60 s) and 20,000 iterations (about 11 h) with/without the method of virtual goals	Task success rate 98.86%
Satheeshbabu et al. (2019)	DQN	3D position reaching	5,000 episodes^a^	3.05 cm
You et al. (2019)	DDQN	3D position reaching	100 episodes	6.58 ± 5.6 mm
Ansari et al. (2017b)	Actor–critic	3D position control	300 episodes	—
Satheeshbabu et al. (2020)	DDPG	3D path tracking	10,000 episodes	≤3 cm
Liu et al. (2020)	PPO	2D tracking with changing goals	6,400 episodes	—

a One episode in reinforcement learning means a sequence of states, actions, and rewards, which ends with the terminal state. The time length of one episode depends on the specific task.

#### 4.2.2 Policy-Based Vs. Value-Based Reinforcement Learning

In addition to the perspective of modeling, reinforcement learning algorithms could also be categorized in terms of the solution of optimal policy: the policy search, value-based, and actor–critic methods (Polydoros and Nalpantidis, 2017; Najar and Chetouani, 2020). Policy search methods could generate optimal policy $\pi^{*}\left(a|s\right)$ parameterized with θ directly using various methods: the gradient-based, sampling-based, and informatic theory methods (Polydoros and Nalpantidis, 2017). In the gradient-based approach, the policy function is maximized with gradient-descent iteratively (Thuruthel et al., 2018; Liu et al., 2020). In contrast to policy search reinforcement learning, value-based methods generate the optimal control policy by optimizing the value function, including SARSA (Ansari et al., 2017b), Q-learning (You et al., 2017; Jiang et al., 2021), DQN (Satheeshbabu et al., 2019; Wu et al., 2020) and its various extensions (e.g., DDQN (You et al., 2019) and Double DQN). The actor–critic approach is a combination of policy-based and value-based reinforcement learning, where the actor executes referring to the policy; thereby the critic calculates the value function to evaluate the actor (Satheeshbabu et al., 2020). Some algorithms (Satheeshbabu et al., 2019; Satheeshbabu et al., 2020; Wu et al., 2020) can be regarded as deep reinforcement learning, which means complex deep neural networks were applied in the control policy, rather than a simple state-action-reward table. This is common in the control for flexible manipulators with high-dimensional action and state variables. Deep reinforcement learning is capable of processing more complicated input formats including images (You et al., 2019) while storing more states and actions, which is particularly significant for continuous control.

## 5 Discussion and Conclusion

In this article, we surveyed the state-of-the-art machine learning–based control strategies of continuum robots. Compared with conventional modeling, learning-based mappings provide effective substitutes for analytical models in the feed-forward control loop, without the need of manual model construction and calibration (note: a brief summary of their comparisons can be found in Table 3). The correction load utilizing the feedback loop is therefore reduced. The format of learning objects also varies a lot; in addition to the direct forward/inverse kinematics/statics relationships, learning-based components also contribute to the optimization or compensation units in several works. For example, in the metaheuristics-assisted approach presented in the study by Amouri et al. (2017), particle swarm optimization (PSO) and the genetic algorithm (GA) were used to solve the optimization of PCC-deduced endpoint coordinates, and the constraints involved obstacle avoidance and tracking trajectories. Some learning-based models focus more on intermediate shape modeling rather than the final goal of end-effector control (Holsten et al., 2019).

**TABLE 3:** A conclusive comparison of analytical-modeling-based and machine-learning-based control.

Aspects		Analytical modeling	Supervised learning*	Reinforcement learning
Human Intervention (Single robot)	Model derivation	✓	✗	✗
	Parameter tuning	✓	✗	✗
	Data collection	✗	✓ (offline)	✓ (online)
	Training	✗	✓	✓
Generalization (Same prototype)	Model derivation	✗	✗	✗
	Parameter tuning	✓	✗	✗
	Data collection	✗	✓ (offline)	✓ (online)
	Training	✗	✓	✓
Dependence on data		Low	high	high
Online refinement		✗	✓	✓
Adaptability to un-modeled disturbances		✗	✓	✓

* Cells marked with gray shading indicate advantages.

All the methods mentioned in Sections 3 and 4 facilitate the release of sensor variety demand. However, for those approaches that solely rely on sensory data, a drawback will be the high requirement on the quality of feedback information (i.e., task space sensing). No matter for iterative- or regression-based machine learning techniques, the distribution and accuracy of sensory data would play an important role, which has been discussed in Section 3.3. To adapt to the unstructured external conditions (e.g., contact forces), online updates should be an emphasis in future applications. This is also one major advantage of learning-based control, while relevant attempts have been made in recent works (Lee et al., 2017a; Ho et al., 2018; Fang et al., 2019). However, for such schemes like the ones in the studies by Yip and Camarillo (2014), Lee et al. (2017a), the question of how to characterize and resolve the outliers with low confidence of the ground truth needs to be carefully considered. Although excessive addition of sensors is not recommended, several self-contained measurement devices such as fiber Bragg gratings (FBGs) which can appropriately accommodate the flexible continuum body have the potential for sensor fusion with positional sensors (Lun et al., 2019; Wang et al., 2020; Wang et al., 2021). Meanwhile, the combination of analytical and data-driven models will be another trend for continuum robot control. Although the analytical approaches encounter challenges in parameter characterization and modeling uncertainties, the convergence of their solutions can usually be guaranteed. Its combination with online learning could leverage both of their respective advantages, which are convergent performance, no prior data exploration, and online control error compensation.

Section 5 concludes the various deployments of reinforcement learning in soft continuum robots and reveals its prospect in dealing with complex learning tasks automatically. Nevertheless, it is observed that most previous works concentrated on simplified tasks such as trajectory tracking and goal reaching, which only take little advantage of the powerful learning tool. Developing the learning ability in sophisticated applications is the main challenge of reinforcement learning in soft robots, even the whole robotics field. Additionally, the effectiveness of interaction data collection also hinders further development. Provided with kinematics/dynamics models, enhancing the validity and efficacy of data collection during environmental interactions will be a significant contribution to reinforcement learning. Without such models, the deviation between simulated and actual manipulators would be a big obstacle for reinforcement learning's application. To handle these cases, Sim-to-Real transfer approaches (Zhao et al., 2020) such as domain randomization and domain adaptation may drive a new research focus in the recent future.
